# FoodNet and Enter-net: emerging surveillance programs for foodborne diseases.

**DOI:** 10.3201/eid0403.980330

**Published:** 1998

**Authors:** S Yang

**Affiliations:** Centers for Disease Control and Prevention, Atlanta, Georgia, USA.


### FoodNet and Enter-net: Emerging Surveillance Programs for Foodborne Diseases

**Samantha Yang**
Centers for Disease Control and Prevention, Atlanta, Georgia, USA

The public health challenges of foodborne diseases are changing as a result of newly identified pathogens and vehicles of transmission, changes in food production and distribution, and an apparent decline in food safety awareness. Response to these new challenges requires new surveillance strategies to monitor the incidence of human illness and provide data for developing effective prevention strategies.

### FoodNet

The Foodborne Diseases Active Surveillance Network (FoodNet), the principal foodborne disease component of the Centers for Disease Control and Prevention's (CDC) Emerging Infections Program (EIP), is a collaborative project with participating EIP sites, the U.S. Department of Agriculture (USDA), and the U.S. Food and Drug Administration (FDA). To determine more precisely the incidence of foodborne illness in the United States, FoodNet was established in five locations (selected counties in California, Connecticut, and Georgia, and the entire states of Minnesota and Oregon) in 1995 and was expanded to selected counties in Maryland and New York in 1997. The population of these seven FoodNet sites in 1997 was 20.3 million (7.7% of the U.S. population).

The objectives of FoodNet are to 1) describe the epidemiology of new and emerging bacterial, parasitic, and viral foodborne diseases of national importance; 2) more precisely determine the frequency and severity of foodborne diseases in the United States; and 3) determine the proportion of foodborne disease caused by eating specific foods. By monitoring foodborne disease incidence over time, FoodNet will document the effectiveness of new food safety initiatives, such as the USDA Food and Safety Inspection Service's Pathogen Reduction and Hazard Analysis and Critical Control Points (HACCP) Rule, in decreasing the number of cases of foodborne disease in the United States each year. To address these objectives, FoodNet conducts active surveillance and related studies: a population survey, a physician survey, and a case-control study of *Escherichia coli* O157:H7 infections.

### Population Survey

Duc Vugia, California Department of Health, reported the results of the FoodNet population survey, which was conducted between July 1, 1996, and June 30, 1997, in selected counties of California, Connecticut, Georgia, and the entire states of Minnesota and Oregon. This survey provided an estimate of the prevalence of acute diarrhea and the frequency with which patients with acute diarrhea sought medical care. In 9,003 interviews, 11% of persons reported acute diarrhea in the 4 weeks before the interview, 12% of persons with acute diarrhea called a health-care provider as a result of this illness, and 8% sought medical care. These data indicate an estimated 1.4 acute diarrheal episodes per person each year in the United States, with 1% of the population seeking medical care because of acute diarrhea.

### Physician Survey

In 1996, to obtain information on stool culturing practices, physicians in California, Connecticut, Georgia, Minnesota, and Oregon were surveyed. Results were reported by Thomas Hennessy, CDC. Of the 1,783 physicians responding to this survey, 44% reported requesting a stool culture from the most recent patient they remembered seeing who had acute diarrhea. Patient factors significantly associated with stool culture requests included bloody stools, a diagnosis of AIDS, diarrhea lasting longer than 3 days, travel to a developing country, and fever. Stool culture ordering practices differed by physicians' specialties and geographic site; however, culturing practices did not differ by payment plan, such as managed care or fee-for-service.

Variability in culturing practices by specialty and geographic location suggests a need for clinical diagnostic guidelines for diarrheal illnesses.

### Case-Control Study

A case-control study was conducted at FoodNet sites to identify risk factors for sporadic *E. coli* O157:H7 infections and to explain variations in the incidence of *E. coli* O157:H7 incidence among FoodNet sites. Heidi Kassenborg, Minnesota Department of Health, presented results from this study, which was conducted between March 1, 1996, and April 30, 1997, in selected counties of California, Connecticut, Georgia, and the entire states of Minnesota and Oregon. Data were obtained from 200 nonoutbreak case-patients and 380 controls, matched by age and telephone exchange. For all sites combined, illness was associated with eating pink hamburgers or pink ground beef and with visiting a farm. Regional variation in beef processing and in exposure to farms may have contributed to the regional variability of *E. coli* O157:H7 infections.

### Enter-net

Funded by the European Commission, Enter-net (formerly Salm-Net) is an international surveillance system for *Salmonella* infections (including data on antibiotic resistance) and *E. coli* O157 infections. Microbiologists and epidemiologists responsible for national laboratory-based surveillance of these pathogens in 15 European countries form the Enter-net network. Ian Fisher, Communicable Disease Control Centre, United Kingdom, described Enter-net and highlighted international outbreaks recognized by the network. Investigations of these outbreaks led to public health interventions and product recalls in Europe. The international database of laboratory-confirmed cases of salmonellosis produced by Enter-net also allows trends in this illness to be observed over several years. For example, Enter-net is documenting the continuing problem of *Salmonella* serotype Enteriditis in western Europe. Enter-net represents a working model of how focused infection-specific international surveillance involving key public health professionals can help monitor and detect international outbreaks of foodborne infection.

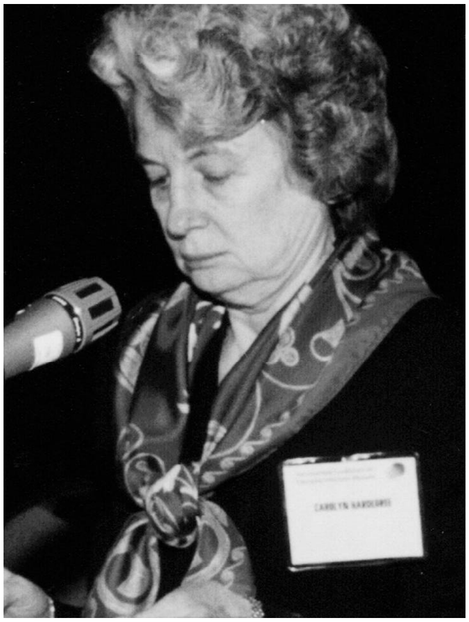

Carol Hardegree
Food and Drug Administration,
Washington, D.C., USA

